# Supplementary material for: Iterative improvement in the automatic modular design of robot swarms
Source: PeerJ Comput Sci. 2020 Dec 7;6:e322. doi: 10.7717/peerj-cs.322 (PMC7924708; doi:10.7717/peerj-cs.322)
Supplement: Supplemental Information 3 [file peerj-cs-06-322-s003.zip › argos3/doc/api/standalone/a00518_source.html]

ARGoS: plugins/robots/generic/simulator/colored\_blob\_omnidirectional\_camera\_rotzonly\_sensor.cpp Source File


- Main Page
- Related Pages
- Namespaces
- Classes
- Files

- File List
- File Members

# plugins/robots/generic/simulator/colored\_blob\_omnidirectional\_camera\_rotzonly\_sensor.cpp

Go to the documentation of this file.

```
00001 #include "colored_blob_omnidirectional_camera_rotzonly_sensor.h"
00002 #include <argos3/core/simulator/simulator.h>
00003 #include <argos3/core/simulator/space/positional_indices/positional_index.h>
00004 #include <argos3/core/simulator/entity/composable_entity.h>
00005 #include <argos3/core/simulator/entity/embodied_entity.h>
00006 #include <argos3/plugins/simulator/entities/led_entity.h>
00007 #include <argos3/plugins/simulator/entities/omnidirectional_camera_equipped_entity.h>
00008 #include <argos3/plugins/simulator/media/led_medium.h>
00009 
00010 namespace argos {
00011 
00012    /****************************************/
00013    /****************************************/
00014 
00015    class COmnidirectionalCameraLEDCheckOperation : public CPositionalIndex<CLEDEntity>::COperation {
00016 
00017    public:
00018 
00019       COmnidirectionalCameraLEDCheckOperation(
00020          CCI_ColoredBlobOmnidirectionalCameraSensor::TBlobList& t_blobs,
00021          COmnidirectionalCameraEquippedEntity& c_omnicam_entity,
00022          CEmbodiedEntity& c_embodied_entity,
00023          CControllableEntity& c_controllable_entity,
00024          bool b_show_rays,
00025          Real f_noise_std_dev) :
00026          m_tBlobs(t_blobs),
00027          m_cOmnicamEntity(c_omnicam_entity),
00028          m_cEmbodiedEntity(c_embodied_entity),
00029          m_cControllableEntity(c_controllable_entity),
00030          m_bShowRays(b_show_rays),
00031          m_fDistanceNoiseStdDev(f_noise_std_dev),
00032          m_pcRNG(NULL) {
00033          m_pcRootSensingEntity = &m_cEmbodiedEntity.GetParent();
00034          if(m_fDistanceNoiseStdDev > 0.0f) {
00035             m_pcRNG = CRandom::CreateRNG("argos");
00036          }
00037       }
00038       virtual ~COmnidirectionalCameraLEDCheckOperation() {
00039          while(! m_tBlobs.empty()) {
00040             delete m_tBlobs.back();
00041             m_tBlobs.pop_back();
00042          }
00043       }
00044 
00045       virtual bool operator()(CLEDEntity& c_led) {
00046          /* Process this LED only if it's lit */
00047          if(c_led.GetColor() != CColor::BLACK) {
00048             if(c_led.HasParent()) {
00049                /* Filter out the LEDs belonging to the sensing entity by checking if they share the same parent entity */
00050                m_pcRootOfLEDEntity = &c_led.GetParent();
00051                while(m_pcRootOfLEDEntity->HasParent()) m_pcRootOfLEDEntity = &m_pcRootOfLEDEntity->GetParent();
00052                if(m_pcRootSensingEntity == m_pcRootOfLEDEntity) {
00053                   return true;
00054                }
00055             }
00056             /* If we are here, it's because the LED must be processed */
00057             m_cOcclusionCheckRay.SetEnd(c_led.GetPosition());
00058             m_cLEDRelativePos = c_led.GetPosition();
00059             m_cLEDRelativePos -= m_cCameraPos;
00060             m_cLEDRelativePosXY.Set(m_cLEDRelativePos.GetX(),
00061                                     m_cLEDRelativePos.GetY());
00062             if(Abs(m_cLEDRelativePos.GetX()) < m_fGroundHalfRange &&
00063                Abs(m_cLEDRelativePos.GetY()) < m_fGroundHalfRange &&
00064                m_cLEDRelativePos.GetZ() < m_cCameraPos.GetZ() &&
00065                !GetClosestEmbodiedEntityIntersectedByRay(m_sIntersectionItem,
00066                                                          m_cOcclusionCheckRay,
00067                                                          m_cEmbodiedEntity)) {
00068                /* If noise was setup, add it */
00069                if(m_fDistanceNoiseStdDev > 0.0f) {
00070                   m_cLEDRelativePosXY += CVector2(
00071                      m_cLEDRelativePosXY.Length() * m_pcRNG->Gaussian(m_fDistanceNoiseStdDev),
00072                      m_pcRNG->Uniform(CRadians::UNSIGNED_RANGE));
00073                }
00074                m_tBlobs.push_back(new CCI_ColoredBlobOmnidirectionalCameraSensor::SBlob(
00075                                      c_led.GetColor(),
00076                                      NormalizedDifference(m_cLEDRelativePosXY.Angle(), m_cCameraOrient),
00077                                      m_cLEDRelativePosXY.Length() * 100.0f));
00078                if(m_bShowRays) {
00079                   m_cControllableEntity.AddCheckedRay(false, CRay3(m_cCameraPos, c_led.GetPosition()));
00080                }
00081             }
00082          }
00083          return true;
00084       }
00085 
00086       void Setup(Real f_ground_half_range) {
00087          while(! m_tBlobs.empty()) {
00088             delete m_tBlobs.back();
00089             m_tBlobs.pop_back();
00090          }
00091          m_fGroundHalfRange = f_ground_half_range;
00092          m_cEmbodiedEntity.GetOriginAnchor().Orientation.ToEulerAngles(m_cCameraOrient, m_cTmp1, m_cTmp2);
00093          m_cCameraPos = m_cEmbodiedEntity.GetOriginAnchor().Position;
00094          m_cCameraPos += m_cOmnicamEntity.GetOffset();
00095          m_cOcclusionCheckRay.SetStart(m_cCameraPos);
00096       }
00097       
00098    private:
00099       
00100       CCI_ColoredBlobOmnidirectionalCameraSensor::TBlobList& m_tBlobs;
00101       COmnidirectionalCameraEquippedEntity& m_cOmnicamEntity;
00102       CEmbodiedEntity& m_cEmbodiedEntity;
00103       CControllableEntity& m_cControllableEntity;
00104       Real m_fGroundHalfRange;
00105       bool m_bShowRays;
00106       CEntity* m_pcRootSensingEntity;
00107       CEntity* m_pcRootOfLEDEntity;
00108       CVector3 m_cCameraPos;
00109       CRadians m_cCameraOrient;
00110       CRadians m_cTmp1, m_cTmp2;
00111       CVector3 m_cLEDRelativePos;
00112       CVector2 m_cLEDRelativePosXY;
00113       SEmbodiedEntityIntersectionItem m_sIntersectionItem;
00114       CRay3 m_cOcclusionCheckRay;
00115       Real m_fDistanceNoiseStdDev;
00116       CRandom::CRNG* m_pcRNG;
00117    };
00118 
00119    /****************************************/
00120    /****************************************/
00121 
00122    CColoredBlobOmnidirectionalCameraRotZOnlySensor::CColoredBlobOmnidirectionalCameraRotZOnlySensor() :
00123       m_bEnabled(false),
00124       m_pcOmnicamEntity(NULL),
00125       m_pcControllableEntity(NULL),
00126       m_pcEmbodiedEntity(NULL),
00127       m_pcLEDIndex(NULL),
00128       m_pcEmbodiedIndex(NULL),
00129       m_bShowRays(false) {
00130    }
00131 
00132    /****************************************/
00133    /****************************************/
00134 
00135    CColoredBlobOmnidirectionalCameraRotZOnlySensor::~CColoredBlobOmnidirectionalCameraRotZOnlySensor() {
00136    }
00137 
00138    /****************************************/
00139    /****************************************/
00140 
00141    void CColoredBlobOmnidirectionalCameraRotZOnlySensor::SetRobot(CComposableEntity& c_entity) {
00142       /* Get omndirectional camera equipped entity */
00143       m_pcOmnicamEntity = &(c_entity.GetComponent<COmnidirectionalCameraEquippedEntity>("omnidirectional_camera"));
00144       /* Get controllable entity */
00145       m_pcControllableEntity = &(c_entity.GetComponent<CControllableEntity>("controller"));
00146       /* Get embodied entity */
00147       m_pcEmbodiedEntity = &(c_entity.GetComponent<CEmbodiedEntity>("body"));
00148    }
00149 
00150    /****************************************/
00151    /****************************************/
00152 
00153    void CColoredBlobOmnidirectionalCameraRotZOnlySensor::Init(TConfigurationNode& t_tree) {
00154       try {
00155          /* Parent class init */
00156          CCI_ColoredBlobOmnidirectionalCameraSensor::Init(t_tree);
00157          /* Show rays? */
00158          GetNodeAttributeOrDefault(t_tree, "show_rays", m_bShowRays, m_bShowRays);
00159          /* Parse noise */
00160          Real fDistanceNoiseStdDev = 0;
00161          GetNodeAttributeOrDefault(t_tree, "noise_std_dev", fDistanceNoiseStdDev, fDistanceNoiseStdDev);
00162          /* Get LED medium from id specified in the XML */
00163          std::string strMedium;
00164          GetNodeAttribute(t_tree, "medium", strMedium);
00165          m_pcLEDIndex = &(CSimulator::GetInstance().GetMedium<CLEDMedium>(strMedium).GetIndex());
00166          /* Create check operation */
00167          m_pcOperation = new COmnidirectionalCameraLEDCheckOperation(
00168             m_sReadings.BlobList,
00169             *m_pcOmnicamEntity,
00170             *m_pcEmbodiedEntity,
00171             *m_pcControllableEntity,
00172             m_bShowRays,
00173             fDistanceNoiseStdDev);
00174       }
00175       catch(CARGoSException& ex) {
00176          THROW_ARGOSEXCEPTION_NESTED("Error initializing the colored blob omnidirectional camera rotzonly sensor", ex);
00177       }
00178    }
00179 
00180    /****************************************/
00181    /****************************************/
00182 
00183    void CColoredBlobOmnidirectionalCameraRotZOnlySensor::Update() {
00184       if(m_bEnabled) {
00185          /* Increase data counter */
00186          ++m_sReadings.Counter;
00187          /* Calculate range on the ground */
00188          CVector3 cCameraPos = m_pcOmnicamEntity->GetOffset();
00189          cCameraPos += m_pcEmbodiedEntity->GetOriginAnchor().Position;
00190          Real fGroundHalfRange = cCameraPos.GetZ() * Tan(m_pcOmnicamEntity->GetAperture());
00191          /* Prepare the operation */
00192          m_pcOperation->Setup(fGroundHalfRange);
00193          /* Go through LED entities in box range */
00194          m_pcLEDIndex->ForEntitiesInBoxRange(
00195             CVector3(cCameraPos.GetX(),
00196                      cCameraPos.GetY(),
00197                      cCameraPos.GetZ() * 0.5f),
00198             CVector3(fGroundHalfRange, fGroundHalfRange, cCameraPos.GetZ() * 0.5f),
00199             *m_pcOperation);
00200       }
00201    }
00202 
00203    /****************************************/
00204    /****************************************/
00205 
00206    void CColoredBlobOmnidirectionalCameraRotZOnlySensor::Reset() {
00207       m_sReadings.Counter = 0;
00208       m_sReadings.BlobList.clear();
00209    }
00210 
00211    /****************************************/
00212    /****************************************/
00213 
00214    void CColoredBlobOmnidirectionalCameraRotZOnlySensor::Destroy() {
00215       delete m_pcOperation;
00216    }
00217 
00218    /****************************************/
00219    /****************************************/
00220 
00221    void CColoredBlobOmnidirectionalCameraRotZOnlySensor::Enable() {
00222       m_pcOmnicamEntity->Enable();
00223       m_bEnabled = true;
00224    }
00225 
00226    /****************************************/
00227    /****************************************/
00228 
00229    void CColoredBlobOmnidirectionalCameraRotZOnlySensor::Disable() {
00230       m_pcOmnicamEntity->Disable();
00231       m_bEnabled = false;
00232    }
00233 
00234    /****************************************/
00235    /****************************************/
00236 
00237    REGISTER_SENSOR(CColoredBlobOmnidirectionalCameraRotZOnlySensor,
00238                    "colored_blob_omnidirectional_camera", "rot_z_only",
00239                    "Carlo Pinciroli [ilpincy@gmail.com]",
00240                    "1.0",
00241                    "A generic omnidirectional camera sensor to detect colored blobs.",
00242                    "This sensor accesses an omnidirectional camera that detects colored blobs. The\n"
00243                    "sensor returns a list of blobs, each defined by a color and a position with\n"
00244                    "respect to the robot reference point on the ground. In controllers, you must\n"
00245                    "include the ci_colored_blob_omnidirectional_camera_sensor.h header.\n\n"
00246                    "REQUIRED XML CONFIGURATION\n\n"
00247                    "  <controllers>\n"
00248                    "    ...\n"
00249                    "    <my_controller ...>\n"
00250                    "      ...\n"
00251                    "      <sensors>\n"
00252                    "        ...\n"
00253                    "        <colored_blob_omnidirectional_camera implementation=\"rot_z_only\"\n"
00254                    "                                             medium=\"leds\" />\n"
00255                    "        ...\n"
00256                    "      </sensors>\n"
00257                    "      ...\n"
00258                    "    </my_controller>\n"
00259                    "    ...\n"
00260                    "  </controllers>\n\n"
00261                    "The 'medium' attribute must be set to the id of the leds medium declared in the\n"
00262                    "<media> section.\n\n"
00263                    "OPTIONAL XML CONFIGURATION\n\n"
00264                    "It is possible to draw the rays shot by the camera sensor in the OpenGL\n"
00265                    "visualization. This can be useful for sensor debugging but also to understand\n"
00266                    "what's wrong in your controller. In OpenGL, the rays are drawn in cyan when\n"
00267                    "they are not obstructed and in purple when they are. In case a ray is\n"
00268                    "obstructed, a black dot is drawn where the intersection occurred.\n"
00269                    "To turn this functionality on, add the attribute \"show_rays\" as in this\n"
00270                    "example:\n\n"
00271                    "  <controllers>\n"
00272                    "    ...\n"
00273                    "    <my_controller ...>\n"
00274                    "      ...\n"
00275                    "      <sensors>\n"
00276                    "        ...\n"
00277                    "        <colored_blob_omnidirectional_camera implementation=\"rot_z_only\"\n"
00278                    "                                             medium=\"leds\" />\n"
00279                    "                                             show_rays=\"true\" />\n"
00280                    "        ...\n"
00281                    "      </sensors>\n"
00282                    "      ...\n"
00283                    "    </my_controller>\n"
00284                    "    ...\n"
00285                    "  </controllers>\n\n"
00286                    "It is possible to add uniform noise to the blobs, thus matching the\n"
00287                    "characteristics of a real robot better. This can be done with the attribute\n"
00288                    "\"noise_std_dev\".\n\n"
00289                    "  <controllers>\n"
00290                    "    ...\n"
00291                    "    <my_controller ...>\n"
00292                    "      ...\n"
00293                    "      <sensors>\n"
00294                    "        ...\n"
00295                    "        <colored_blob_omnidirectional_camera implementation=\"rot_z_only\"\n"
00296                    "                                             medium=\"leds\" />\n"
00297                    "                                             noise_std_dev=\"0.1\" />\n"
00298                    "        ...\n"
00299                    "      </sensors>\n"
00300                    "      ...\n"
00301                    "    </my_controller>\n"
00302                    "    ...\n"
00303                    "  </controllers>\n",
00304                    "Usable"
00305                   );
00306 
00307 }
```

---

Generated on 10 Jul 2018 for ARGoS by 
 1.6.1
